# Impact of *Enterobius vermicularis* infection on biochemical parameters in the blood of children in Erbil Province, Iraq

**DOI:** 10.1186/s12879-020-05064-w

**Published:** 2020-05-12

**Authors:** Ahmed Akil Khudhair Al-Daoody, Eman Najdy Haydar Al-Bazzaz

**Affiliations:** 1 Department of Medical Microbiology, College of Health Sciences, Hawler Medical University, Erbil, Kurdistan Region Iraq; 2 Department of Medical Laboratory Techniques, Erbil Health technical College, Erbil Polytechnic University, Erbil, Iraq

**Keywords:** *Enterobius vermicularis*, Vitamin B12, Folate, IgE, Biochemical aspects, Children

## Abstract

**Background:**

*Enterobius vermicularis* is an intestinal helminthic parasite that causes a gastrointestinal infection called enterobiasis. Children are more susceptible to infection than adults. The current study aimed to explore the prevalence of *E. vermicularis* infection among children in Erbil City concerning demographic factors and certain blood parameters.

**Methods:**

A cross-sectional and analytical study was conducted on 505 children (3–10 years). Cellophane tape samples and blood samples were taken from participants. The cellophane tape samples were examined microscopically, whereas blood samples were examined using the auto-analyzer and Cobas.

**Results:**

The overall prevalence of *E. vermicularis* infection was 27.13%, and the infection rate was non-significantly (*P* = 0.371) higher in females (28.85%) than in males (25.31%). The incidence of enterobiasis was directly proportional to family size. This study demonstrated that the mean serum total protein and iron levels were significantly decreased in infected children, while other trace element levels were not significantly affected.

**Conclusions:**

The prevalence of *E. vermicularis* is relatively lower than that in previous studies. Serum total protein and iron levels significantly decreased in the enterobiasis-positive group.

## Background

*Enterobius vermicularis* (pinworm, threadworm or seatworm) is one of the helminths that has a larger geographical distribution than other helminths [1]. It has been estimated to infect at least 200 million people worldwide, particularly in children [2, 3]. An outbreak of *E. vermicularis* infection is associated with contaminated food, improper environmental sanitation and inadequate personal hygiene [4].

Parasites are dependent on their host for survival and require nutrients to carry out essential functions including reproduction and growth. Essentially, the nutrients required from the host are carbohydrates, amino acids and lipids [5]. The normal proliferation of cells depends on adequate folate and vitamin B12; they play an important role in DNA synthesis and neurologic function [6]. Additionally, phosphorus has an essential role in the synthesis of ATP, DNA, RNA and cell membrane structures [7]. Although childhood zinc, copper, magnesium and vitamin B12 deficiencies are rather unusual, recent studies have suggested that several intestinal parasites may produce these deficiencies [8, 9]. Early detection and treatment of intestinal parasitic infection are very important to obtain optimal levels of growth, development, immune response and intellectual capacity [10].

The marked eosinophilia and raised immunoglobulin E (IgE) levels in helminth infected patients suggest a large worm load with possible tissue invasion [11]. IgE is an antibody that plays an important role in allergies, but the clinical appearance of immediate hypersensitivity responses is relatively rare in helminthic infections, although a high level of IgE is commonly seen in infected individuals [12].

The current study was carried out to fulfil the following objectives:

1. To study the prevalence of *E. vermicularis* infestation among children, age, gender, and family size.
2. To investigate the possible association of enterobiasis and levels of vitamin B12, IgE, folate, total protein, magnesium, phosphor, and iron in the blood.

## Methods

### Study duration and samples

A cross-sectional study was performed in Erbil City from November 2013 to the end of August 2014. A total of 505 children participated in this study, and their ages ranged between 3 and 10 years old, and included both sexes (245 males and 260 females). The chosen areas for sampling included children from all socioeconomic levels to determine the true prevalence of the infection. A special questionnaire form was prepared for each participant child in the study and filled out by interviewing their mothers.

### Cellophane tape sample collection and examination

The samples were collected by pressing the sticky side of the tape several times on the anal and perianal region of the children and then sticking the tape on the labelled glass slide and putting it in a sterile clean nylon envelope and then enclosing it tightly. This method was carried out with the help of the children’s mothers at night or in the early morning before defecation, using the toilet or taking bath [1, 13]. The collected samples were transported to the Microbiology Laboratory, College of Medicine, Hawler Medical University, and examined under a light microscope (1000x).

### Blood sample collection and examination

Four millilitres of venous blood was taken from 92 children, 67 of whom were infected (enterobiasis-positive group) and 25 of whom were non-infected (enterobiasis-negative group). Samples were collected at Raperin Paediatric Teaching Hospital.

The blood samples were collected in non-heparinized test tubes as described by [14] and transported to the Microbiology Laboratory of the College of Medicine, and they were centrifuged at 3000 rpm for 5 min. Then, the serum was separated and transferred to two Eppendorf tubes, one of which was directly examined by a Biolis 24i Autoanalyser for estimation of the levels of total protein, phosphorus, iron, and magnesium, while the other Eppendorf tube was stored at − 20 °C until examination by Cobas E 411 for evaluation of the levels of vitamin B12, folate, and IgE.

### Ethical considerations

The study was approved by the Research Ethics Committee of the College of Health Sciences - Hawler Medical University. A supporting official letter was taken from Hawler Medical University to the principals of foundations where the study sampling was done. Written permissions were obtained from the principal of foundations and verbal permission was obtained from parents of participating children. Information was given to the parents about the study, method of sampling, disease and its importance for the children’s health, and then they were informed about the results of the tests. Assurance was given to the parents that the obtained information will be kept only for the purpose of the study and not be used in any other purpose apart from the study.

### Data entry and statistical analysis

The collected data were coded using a specially designed coding system and entered into the Statistical Package for Social Sciences (SPSS) version 22.0. The association between the two variables in this study was analysed and assessed using the chi-square test. Frequency analysis was used to describe counting numbers and percentages of different variables. The t-test was used to investigate the difference between the two groups according to serum parameters. *P* ≤ 0.05 was considered statistically significant.

## Results

### Prevalence of *Enterobius vermicularis* infection among children according to sex in Erbil City

**Fig. 1:**
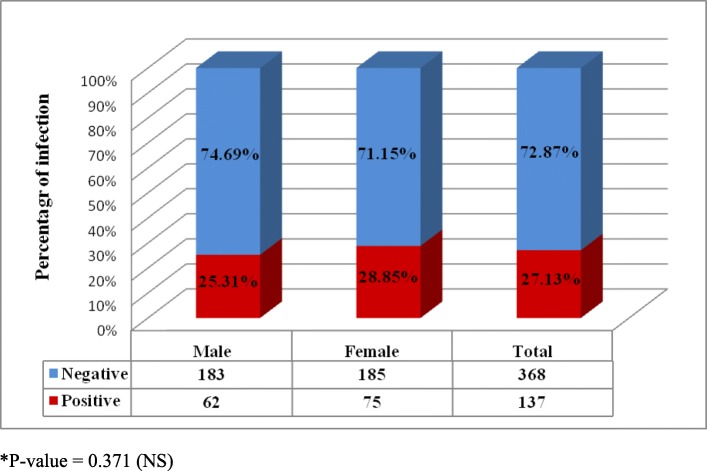
Prevalence of *Enterobius vermicularis* among children in relation to sex. *P-value = 0.371 (NS)

As illustrated in Fig. 1, the total rate of infection with *E. vermicularis* among 505 children was 27.13% (137/505) using the cellophane tape method. However, the frequency of infection among females was higher than that among males at rates of 28.85% (75/260) and 25.31% (62/245), respectively. Statistically, no significant (*P* > 0.05) difference in infection rate was recorded between males and females with enterobiasis.

### Prevalence of *E. vermicularis* infection according to age group

The highest rate of infection was recorded at 27.42% (65/237) in the younger age group (≤ 6 years), while the lowest was recorded at 26.86% (72/268) in children aged 7 years and above. Statistically non-significant (P > 0.05) differences were observed among age groups concerning enterobiasis (Table 1).

**Table 1:** Prevalence of *Enterobius vermicularis* among children in relation to age group

Age groups	Total no. of examinations	Gender	No. of enterobiasis -ve	No. of enterobiasis + ve	Total no. (%) of enterobiasis + ve
**3–6**	**237**	Male	83	29	**65 (27.42%)**
		Female	89	36	
**7–10**	**268**	Male	100	33	**72 (26.86%)**
		Female	96	39	
**Total**	**505**		368	137	**137 (27.13%)**
***P*-value**	**0.888 (NS)**				

### Prevalence of *E. vermicularis* infection in relation to family size

As seen in Table 2, the infection rate is directly proportional to the family size, as the lowest rate of infection, 18.3% (15/82), was in children from small families (4 persons), and the rate of infection increased gradually by increasing the family size to 83.3% (5/6) in children from large families (12 persons). Statistically, it was found that family size is non-significantly (*P* > 0.05) correlated with pinworm infection, based on the chi-square test.

**Table 2:** **Relationship between*Enterobius vermicularis*infection and family size**

Family size	2	3	4	5	6	7	8	9	10	11	12	13	15	16
Enterobiasis														
**Total no. of Examinations**	**2**	**22**	**82**	**143**	**104**	**66**	**33**	**21**	**19**	**3**	**6**	**2**	**1**	**1**
**No. -ve**	**1**	**15**	**67**	**102**	**78**	**47**	**23**	**13**	**14**	**3**	**1**	**2**	**1**	**1**
**No. +ve**	**1**	**7**	**15**	**41**	**26**	**19**	**10**	**8**	**5**	**0**	**5**	**0**	**0**	**0**
(%) + ve Enterobiasis of each family size*	**50**	**31.8**	**18.3**	**28.7**	**25**	**28.9**	**30.3**	**38.1**	**26.3**	**0**	**83.3**	**0**	**0**	**0**

*P-value = 0.369 (NS)

### Effect of *E. vermicularis* infection on blood parameters

The results of blood parameters concerning enterobiasis are presented in Table 3. The mean values of serum total protein, phosphorus, iron, magnesium, vitamin B12; folate and IgE of 67 children infected with *E. vermicularis* were compared with those of 25 non-infected children.

**Table 3:** Mean serum levels of some biochemical parameters in *Enterobius vermicularis* infection

Serum parameters	Means and Std. Deviations of Enterobiasis –ve	Means and Std. Deviations of Enterobiasis + ve	***P***-value	Statistical Analysis
**Total protein (g/dl)**	**7.65 ± 0.61**	**7.06 ± 0.64**	**0.0008**	**VHS**
**Phosphorus (mg/dl)**	**3.75 ± 0.79**	**4.07 ± 0.76**	**0.077**	**NS**
**Mg (mg/dl)**	**1.88 ± 0.31**	**1.83 ± 0.39**	**0.583**	**NS**
**Iron (μg/dl)**	**86.40 ± 42.59**	**68.74 ± 32.01**	**0.036**	**S**
**VB12 (pg/mL)**	**631.88 ± 271.34**	**507.16 ± 256.91**	**0.055**	**NS**
**Folate (ng/mL)**	**13.95 ± 3.87**	**12.45 ± 4.05**	**0.128**	**NS**
**IgE (IU/mL)**	**96.17 ± 129.01**	**170.26 ± 216.28**	**0.119**	**NS**

The results show that the mean value of serum total protein was significantly (*P* < 0.001) lower in the infected group, and the serum iron level was decreased in the infected group. Statistical analysis showed that there was a significant (*P* < 0.05) difference between both groups; on the other hand, levels of phosphorus, magnesium, vitamin B12, folate, and IgE varied in the comparison between the enterobiasis-positive and enterobiasis-negative groups, but there was no statistically significant (*P* > 0.05) difference between them.

## Discussion

Children are the most susceptible group of any population that can be infected by the parasites. They could also be asymptomatic carriers which may pose heightened danger and constitute particularly public health problems [15].

The prevalence of pinworm infection was 27.13% (137) among 505 children using the cellophane tape method. Our result was consistent with the prevalence of 29.8% in Erbil City recorded by [16] among primary school children and with the 29.4% prevalence rate reported by [17] in Turkey (Antakya) among children.

The prevalence observed in this study was lower than the 52.3% recorded by [18] among 2 to 12-years-old children in Baghdad and 86% in Turkey (Ankara) among 6 to 12-years-old children [19]. However, the result of this study was higher than the prevalence of 6.2% in Sri Lanka (Hambanatona district) [20], 7.1% in Iran (Mazandaran) [21], and 0.6% in Birbir town, southern Ethiopia [22].

The difference in the prevalence of *E. vermicularis* infection in different areas is often believed to be related to the age of children, behavioural patterns and area of the bedroom [23]. Personal hygiene, educational level among children, overcrowding, socioeconomic condition, and climate could be related to pinworm infection [24].

The relationship between the prevalence of *E. vermicularis* and sex in the current study showed that the risk of *E. vermicularis* infection was higher but not significant among female children using the adhesive tape method. These data support the findings other studies that have reported a higher prevalence of this helminth among female children: [15] in Mosul City and [25] in the Al- Mahmoudyia area in Baghdad, while in contrast to the present study, other studies reported a higher prevalence among male children [26] in Basrah and [27] in Egypt (Damietta governorate).

Although the infection rate among age groups in both infected and non-infected groups did not differ significantly, the highest rate was 27.42% in the younger age group (3–6 years old). This finding was supported by the reports of other studies done by [28] in Babylon Governorate and [29] in China (Guangdong). This finding was in contrast with those obtained by [30] in Sulaymaniy Governorate and [31] in Northern Thailand.

This difference in the rate of infection among age groups may be because the awareness of attention to personal hygiene is much lower among the small children in comparison to older children, as they spend more time outside the house. Therefore, they have more chances to play with dirt and have a greater frequency of physical contact with their friends than do younger children; thus, they have a higher risk of acquiring pinworm infection [3]. Additionally, the anal scratching habit (by the way hand-mouth transmission) is more frequent among small children, facilitating the autoinfection mode of transmission.

In relation to family size, the infection rate among children belonging to large families (12 persons) was 83.3%, i.e., higher than those found in smaller families. This result is in agreement with that found by [32] in Misan [33] in the Philippines (Albay), who reported that the rate of enterobiasis among children who belong to large families was higher than that of smaller families, so the infection among children increases with increasing family size.

Although enterobiasis is transmitted from one person to another directly and does not require any intermediate host, it is more likely to spread among members of the same family, especially overcrowded families. In addition, clinically mild cases and asymptomatic infected individuals may provide a hidden reservoir of infection in the family population [34]. Moreover, by increasing the number of family members, the possibility of bringing the infection from outside to inside of the home increases.

Immunoglobulin E has an important role in immune defence against most parasitic worms [35], and many researchers have observed a high level of IgE in parasitic infections [8, 36]. The present study found that patients infected with *E. vermicularis* had no significantly higher levels of serum total IgE in comparison with the enterobiasis-negative group. The serum total IgE level in the present study was in agreement with that recorded by [37] in Egypt and [36] in Turkey.

On the other hand, the mean serum vitamin B12 and folate levels in the enterobiasis-positive group were not significantly lower than those in the enterobiasis-negative group. These results were in accordance with those recorded by [14] in Spain and [38] in Iran (Kashan). The deficiency of vitamin B12 and folate are related to anaemia and cell divisions. Additionally, vitamin B12 is involved in the synthesis of DNA [6]. Several parasites inhabit the intestine and cause damage to the intestinal epithelium. Therefore, these parasites may lead to malabsorption and cause vitamin B12 and folate deficiency [39].

Intestinal parasites use food sources of the host, such as carbohydrates, minerals, vitamins and lipids, as essential energy sources for their life cycle. Minerals play an important role in the biochemical and physiological processes of the human body, particularly in growing children. Many studies have recorded that minerals such as magnesium, iron, copper, and zinc have a vital role in the growth and development of children [40, 41].

The mean serum phosphorus level in the enterobiasis-positive group was not significantly higher than that of the enterobiasis-negative group, and the serum phosphorus level in the present study was similar to that recorded by [42] in northern India.

The mean serum total protein and serum iron levels in the enterobiasis-positive group were significantly lower than those in the enterobiasis-negative group. This result is similar to that recorded by other investigators [43, 44]. Although the mean value of serum magnesium level decreased in the enterobiasis-positive group, statistically there was no significant difference between the enterobiasis-positive and enterobiasis-negative groups, which coincides with results found by [45] in Iran. Generally, the decrease in serum trace element levels is probably due to the loss of appetite that can be caused by pinworm infection.

## Conclusions

The present study indicates that *E. vermicularis* is one of the common causative agents of intestinal parasitic infections among children in Erbil City and may alter the normal values of biochemical parameters in the blood of infected children.

## Data Availability

The datasets used for the current study are available on reasonable request. Please contact the corresponding author, Ahmed Akil Khudair Al-Daoody, for data requests.

## Abbreviations

DNA: Deoxyribonucleic acid
RNA: Ribonucleic acid
ATP: Adenosine tri-phosphate
IgE: Immunoglobulin E
